# Prevalence and Associations of Illicit Drug and Polydrug Use in People Living with HIV in Vienna

**DOI:** 10.1038/s41598-018-26413-5

**Published:** 2018-05-23

**Authors:** Igor Grabovac, Michael Meilinger, Horst Schalk, Birgit Leichsenring, Thomas Ernst Dorner

**Affiliations:** 1 Department of Social and Preventive Medicine, Centre for Public Health, Medical University of Vienna, Kinderspitalgasse 15/1, 1090 Vienna, Austria; 2 2nd Department of Respiratory and Critical Care, Otto Wagner Hospital, Baumgartner Höhe 1, 1140 Vienna, Austria; 3 “Schalk - Pichler” Group-practice, Zimmermannplatz 1, 1090 Vienna, Austria; 4 AIDS Hilfe Wien, Mariahilfer Gürtel 4, 1060 Vienna, Austria

## Abstract

We aimed to determine the prevalence of drug and polydrug use in people living with HIV in Austria for the first time for which a two center cross-sectional study was performed. Participants were recruited from consecutive patients during their regularly scheduled visits. In total 438 participants were included in the analysis. For this study we used paper-pencil and online-based questionnaires. The prevalence of illicit drug use was 60.5%; with cannabis use at 31.5%, nitrates at 31.5%, sildenafil/tadalafil at 24% and cocaine at 14%, being the most used substances. Use of more than one substance (polydrug) in drug users was 69.4% or 42.0% in the total study population. Younger age, male gender, and living in an urban area were associated with drug use. Moreover, drug use during clubbing and sex, HIV therapy non-adherence and younger age were associated with polydrug use. Drug users reported condomless sex in 42.4% and performing sexual acts they would not do sober in 44.1%. Results indicate a high prevalence of illicit drug use in PLWHIV in Austria. New research focusing on illicit drug use in PLWHIV should focus on the use of substances during sex and surrounding practices.

## Introduction

With the development of antiretroviral therapy (ART) the mortality and morbidity associated with HIV/AIDS has decreased, even as HIV still poses a major global public health concern with 1.8 million newly diagnosed cases in 2016^1^. This reduction in mortality was brought about by newer antiretroviral therapy with improved tolerability and led to HIV being considered a chronic illness^2^. This paradigm shift calls for a more tailored approach to people living with HIV (PLWHIV) as emerging issues such as healthy aging and non-AIDS conditions are gathering more importance^2,3^. Moreover, issues of addiction seem to be especially problematic within PLWHIV as multiple studies show a two to three fold higher prevalence of tobacco smoking in comparison to the general population, which is often connected to use of alcohol and illegal drugs^4–7^.

Additional to tobacco and alcohol use, illegal drug use is a cause of major harm for societies and individuals^8^. According to the European Drug Report 2017, 26.3% of the European population tried cannabis at least once, with additional estimates of 5.2% for cocaine, 4.2% for 3,4-Methylenedioxymethamphetamine (MDMA) and 3.8% for amphetamines, with injecting drug use showing a decline^9^. The importance of drug use and its connection to HIV is therefore shifting from the “traditional” issues of intravenous drug users and the associated HIV transmission towards illicit drug use that, although underinvestigated, seem to be of high prevalence in certain “key populations” such as men who have sex with men (MSM). The EMIS-Study in Europe showed high prevalence of drugs associated with “chemsex” (a term describing sexual relations under the influence of various, mostly psychoactive substances) including amyl nitrates (“poppers”) and cannabis^10,11^. In terms of HIV and sexually transmitted infections (STIs), illicit drug use and chemsex may be linked to an increase in incidence as studies show that drug use is associated with an increase in condomless sex, including among HIV-serodiscordant partners^10,12–14^. More importantly, studies suggest that MSM who are living with HIV are more likely to use almost all types of illicit drugs in comparison to HIV negative MSM^15,16^. An even more considerable problem among MSM living with HIV is the high prevalence of polydrug use (use of more than one substance within a time period), which was also associated with condomless sex with multiple new partners^11,17,18^. Furthermore, results from the ASTRA study showed an association with increasing polydrug use and increasing frequency of condomless sex^19^. Additionally, methamphetamine use and to a lesser extent use of sildenafil have been shown to be associated with increased high-risk sexual behavior in both MSM and heterosexual samples^17^.

However, problems of illicit drug use go beyond the increased risk for condomless sex and may have relevant clinical consequences for PLWHIV who are on ART due to adherence problems. A particularly high adherence level is necessary to avoid resistance against antiretrovirals and keep the effectiveness of ART in desired levels^20^. Furthermore, illicit drug use has been found to be a factor of non-adherence by causing temporary cognitive impairment or by drug-drug interaction causing potentially toxic side effects, on the other hand also limiting the effectiveness due to shared metabolic pathways^21,22^. Illicit drug use, especially cocaine, amphetamines, methamphetamines, heroin and morphine have been also known to increase the HIV replication and cause epigenetic changes in brain tissue, which can further have a synergistic effect and accelerates neural injury and cognitive impairment^23–25^.

Most studies dealing with the issues of illicit drug use in PLWHIV are solely focused on the MSM population. A recent review on the recreational drug use in PLWHIV in Europe included 13 studies, however only two studies investigated PLWHIV in general (without any additional factors such as specific comorbidities or use of the same medication)^26^. The same review noted high use of recreational drugs in Europe with even higher prevalence in PLWHIV as well as more use of new drugs (such as ketamine, mephedrone, gamma-hydroxybutyrate (GHB), etc.) and those associated with sexual practices.

In terms of prevalence of drug use in the general adult population, Austria is in the mid to lower part of the EU range, with use in 2015 being reported for cannabis at 6.4%, 0.4% for cocaine, MDMA and amphetamines respectively^9^. Long term analysis shows a slight increase in cannabis use, however in general the use of illegal drugs in Austria is concentrated among young adults (aged 15–34) with males reporting higher prevalence^9^. Prevalence of illicit drug use among PLWHIV in Austria has not been investigated so far. The primary aim of this study was to describe the prevalence of illicit drug and polydrug use in a sample of adult PLWHIV and their associations with socio-demographic, HIV related and lifestyle factors.

## Results

A total of 683 patients matched inclusion criteria and 452 (66.1%) agreed to participate. Response rate varied between the centers with center “A” reaching 72.6% (318 approached and 231 agreed) and “B” reaching 60.5% (365 envelopes given and 221 filled out). Further 14 questionnaires needed to be eliminated from analysis as nine filled out less than 50% of the needed questions, four indicated they were not HIV positive and one indicated being 17 years old, leaving 438 participants available for analysis. Significant differences were found between study centers, where center “A” had older participants (45.80 (12.03) v 41.24 (9.66); p < 0.001) with more years passed since HIV diagnosis (13.81 (8.35) v 9.89 (7.40); p < 0.001). In center “B” there were significantly more participants who used drugs (71.1% v 50.0%; p < 0.001) as well as polydrug users (74.2% v 62.7%; p = 0.046).

Our sample consisted of predominantly male, self-identifying homosexual men of high educational level living in urban areas. The sample was stratified by drug use over the past 6 months, where 39.5% (173) participants were classified as non-drug users and 60.5% (265) as drug users. There were significant differences between non-drug and drug users observed in age, sex, sexual orientation, place of residence, mean duration of HIV status and mean duration of ART. Participants classified as drug users were further categorized as monodrug (30.6%; 81) or polydrug (69.4%; 184) users based on the number of drugs they indicated taking within the last 6 months. Significant differences were observed only in age and duration of HIV status (Table 1).

**Table 1:** Sociodemographic and HIV related characteristics of the study population stratified by drug and polydrug^a^ use.

Variable	Total (N = 438)	Non drug users (n = 173)	Drug users (n = 265)	p	Monodrug users (n = 81)	Polydrug users (n = 184)	p
Mean age in years (SD)	43.53 (11.14)	46.87 (11.70)	41.35 (10.21)	<0.001	45.05 (11.40)	39.72 (9.20)	<0.001
Gender				<0.001			0.122
Male % (n)	86.8 (380)	79.8 (138)	91.3 (242)		87.7 (71)	92.9 (171)	
Female % (n)	12.3 (54)	20.2 (35)	7.2 (19)		11.1 (9)	5.4 (10)	
Sexual Orientation				0.001			0.785
Heterosexual % (n)	27.4 (120)	37.0 (64)	21.1 (56)		23.5 (19)	20.1 (37)	
Bisexual % (n)	10.3 (45)	8.7 (15)	11.3 (30)		9.9 (8)	12.0 (22)	
Homosexual % (n)	62.3 (273)	54.3 (94)	67.5 (179)		66.7 (54)	67.9 (125)	
Current relationship				0.143			0.064
Yes % (n)	53.4 (234)	57.8 (100)	50.6 (134)		40.7 (33)	53.3 (98)	
No % (n)	46.6 (204)	42.2 (73)	49.4 (131)		59.3 (48)	46.7 (86)	
Country of birth				0.662			0.133
Austria % (n)	83.1 (364)	83.8 (145)	82.6 (219)		86.4 (70)	81.0 (149)	
EU member state % (n)	8.0 (35)	7.5 (13)	8.3 (22)		2.5 (2)	10.9 (20)	
Non EU state % (n)	5.3 (23)	4.0 (7)	6.0 (16)		7.4 (6)	5.4 (10)	
Outside of Europe % (n)	3.7 (16)	4.6 (8)	3.0 (8)		3.7 (3)	2.7 (5)	
Residence				0.032			0.427
Community up to 5000 residents % (n)	9.1 (40)	13.3 (23)	6.4 (17)		9.9 (8)	4.9 (9)	
Town up to 100000 residents % (n)	6.6 (29)	8.7 (15)	5.3 (14)		4.9 (4)	5.4 (10)	
Large town up to million residents % (n)	6.8 (30)	6.9 (12)	6.8 (18)		4.9 (4)	7.6 (14)	
City with more than a million residents % (n)	77.4 (339)	71.1 (123)	81.5 (216)		80.2 (65)	82.1 (151)	
Highest level of education				0.961			0.061
Primary education % (n)	8.2 (36)	7.5 (13)	8.7 (23)		9.9 (8)	8.2 (15)	
Vocational education % (n)	35.6 (156)	37.6 (65)	34.3 (91)		44.4 (36)	29.9 (55)	
Secondary education % (n)	22.4 (98)	22.0 (38)	22.6 (60)		22.2 (18)	22.8 (42)	
Tertiary education % (n)	31.7 (139)	31.2 (54)	32.1 (85)		23.5 (19)	35.9 (66)	
No formal education % (n)	2.1 (9)	1.7 (3)	2.3 (6)		0	3.3 (6)	
Employment status				0.307			0.711
Full time % (n)	56.4 (247)	58.4 (101)	55.1 (146)		55.6 (45)	54.9 (101)	
Part time % (n)	16.7 (73)	13.3 (23)	18.9 (50)		16.0 (13)	20.1 (37)	
Unemployed % (n)	26.9 (118)	28.3 (49)	26.0 (69)		28.4 (23)	25.0 (46)	
*HIV related variables*							
Mode of HIV transmission				0.086			0.822
Sexual contact % (n)	78.8 (345)	74.6 (129)	81.5 (216)		80.2 (65)	82.1 (151)	
Intravenous drug use % (n)	5.7 (25)	4.6 (8)	6.4 (17)		8.6 (7)	5.4 (10)	
Blood transfusion % (n)	1.8 (8)	2.3 (4)	1.5 (4)		1.2 (1)	1.6 (3)	
Not clear % (n)	13.5 (59)	18.5 (32)	10.2 (27)		9.9 (8)	10.3 (19)	
Time passed since HIV diagnosis in years (SD)	11.85 (8.13)	13.67 (8.44)	10.66 (7.70)	<0.001	12.26 (8.41)	9.97 (7.29)	0.038
Current CD4+ count known				0.333			0.076
Yes % (n)	79.7 (349)	82.1 (142)	78.1 (207)		85.2 (69)	75.0 (138)	
No % (n)	20.3 (89)	17.9 (31)	21.9 (58)		14.8 (12)	25.0 (46)	
Currently on ART				0.253			0.670
Yes % (n)	431 (98.4)	99.4 (172)	97.7 (259)		98.8 (80)	97.3 (179)	
No % (n)	7 (1.6)	0.6 (1)	2.3 (6)		1.2 (1)	2.7 (5)	
Mean duration of therapy in years (SD)	9.84 (6.98)	11.15 (7.23)	8.96 (6.67)	0.002	9.77 (7.25)	8.59 (6.38)	0.194
Number of different ART				0.701			0.607
One % (n)	57.1 (250)	59.3 (102)	57.1 (148)		57.5 (46)	57.0 (102)	
Two % (n)	31.3 (137)	32.0 (55)	31.7 (82)		28.8 (23)	33.0 (59)	
More than 3% (n)	10.0 (44)	8.7 (15)	11.2 (29)		13.8 (11)	10.1 (18)	
Antiretroviral class							
Fusion inhibitors % (n)	0.7 (3)	0.6 (1)	0.8 (2)	1.000	0	1.1 (2)	0.571
Protease inhibitors % (n)	13.0 (56)	11.6 (20)	13.9 (36)	0.560	16.3 (13)	12.8 (23)	0.560
Integrase inhibitors % (n)	20.8 (91)	21.5 (37)	20.8 (54)	0.904	20.0 (16)	21.2 (38)	0.870
Reverse transcriptase inhibitors % (n)	62.3 (273)	62.8 (108)	63.7 (165)	0.919	60.0 (48)	65.4 (117)	0.485
Fixed combinations (integrase and reverse transcriptase inhibitors) % (n)	33.1 (145)	33.1 (57)	34.0 (88)	0.917	37.5 (30)	32.4 (58)	0.478

### Drug use

**Figure 1:**
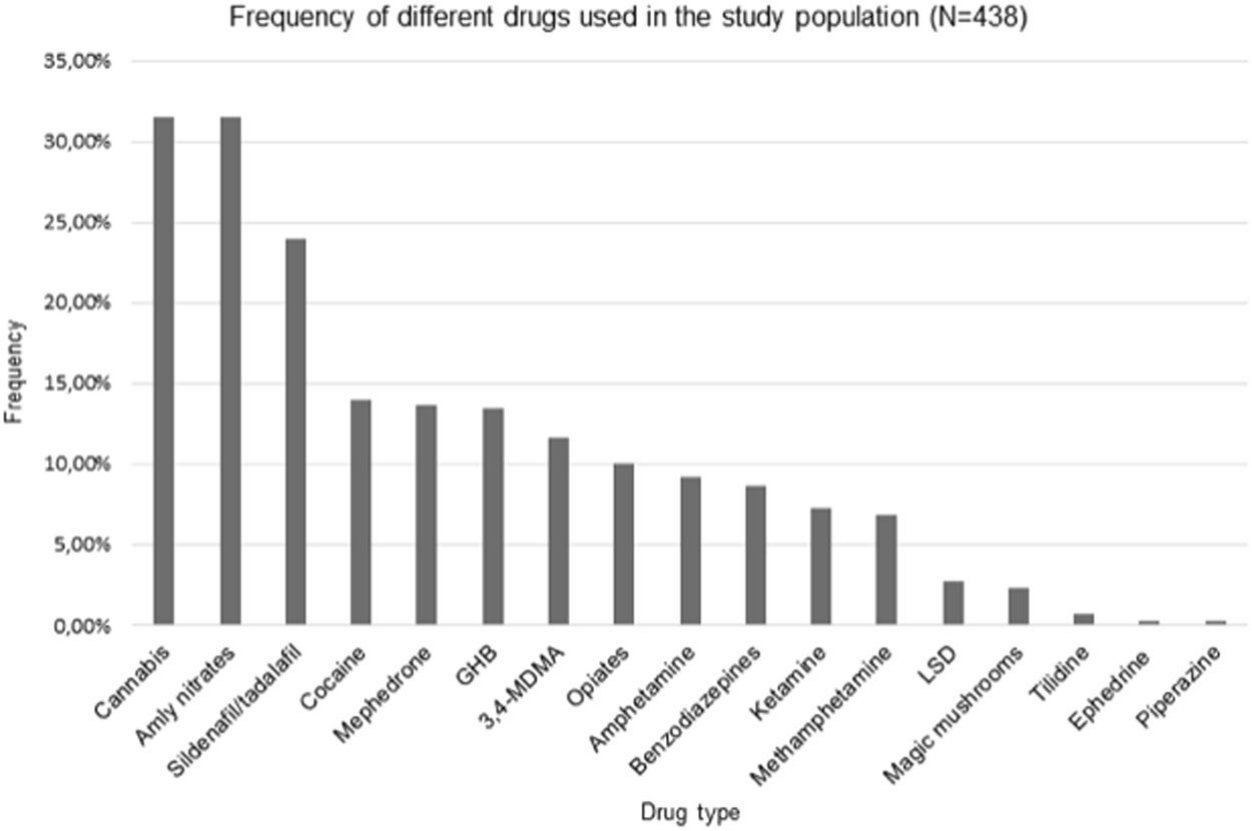
Frequency of different drugs used in the study population (N = 438). A multiple-choice question. Sildenafil/tadalafil and opiod use are based on reported use without a prescription. 3,4-MDMA = 3,4-Methylenedioxymethamphetamine; LSD = Lysergic acid diethylamide; GHB = gamma-Hydroxybutyric acid.

**Figure 2:**
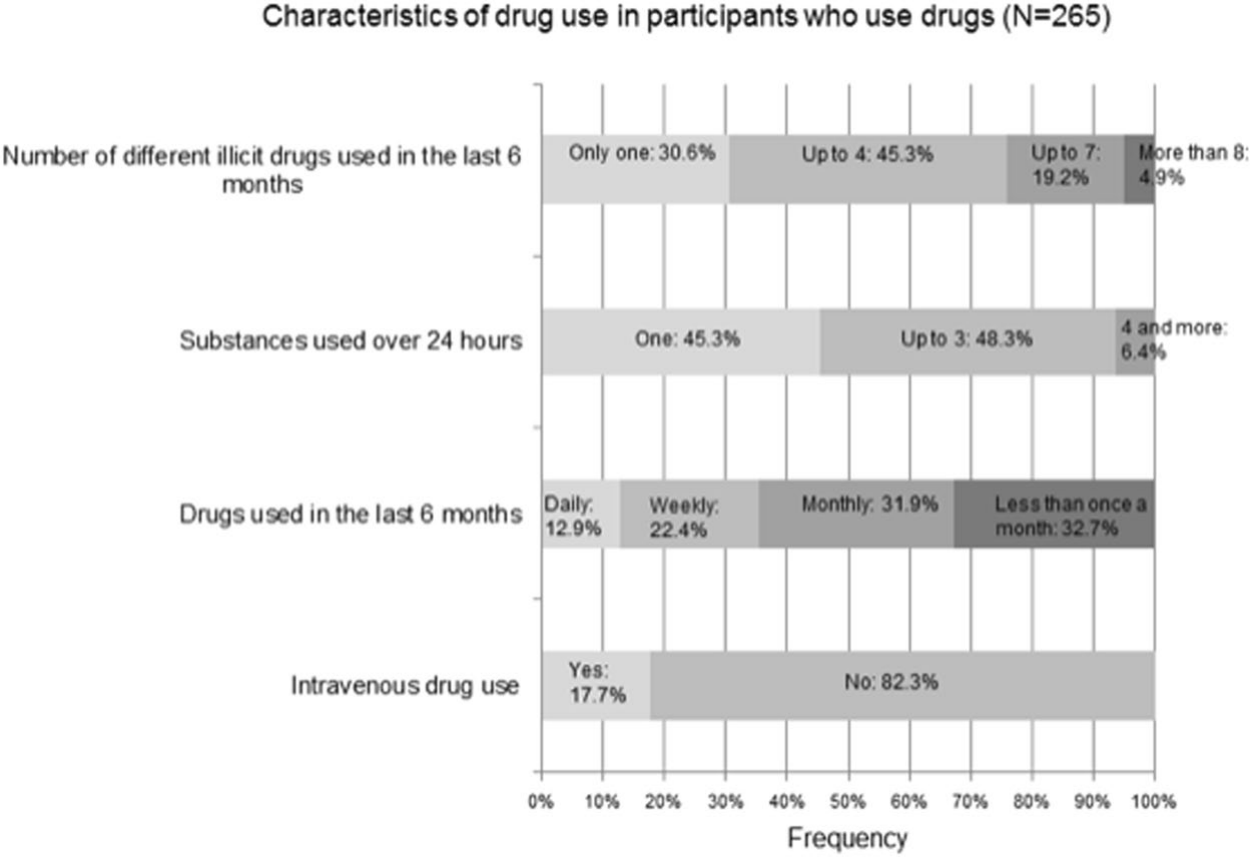
Characteristics of illicit drug use among participants who use drugs (N = 265).

Participants classified as drug users mostly used up to 4 different substances in the past 6 months. Illicit drugs were used less frequently with most of the sample indicating use on a less than monthly and monthly basis. Most used drugs were cannabis and amyl nitrates (“poppers”) followed by sildenafil/tadalafil and cocaine, with the most frequent reason being “for more sexual stimulation”. Accordingly, most drugs were used at home or during sex. Information on drug use can be found in Table 2 and Figs 1 and 2.

**Table 2:** Illicit drug use characteristic among participants that reported using drugs (N = 265).

Variable	Result
Reasons for taking drugs^a^	
To feel happy and energetic % (n)	36.2 (96)
To feel calm % (n)	50.9 (135)
To have more fun % (n)	45.7 (121)
For more sexual stimulation % (n)	72.8 (193)
To forget about worries % (n)	24.5 (65)
To feel more selfworth % (n)	13.6 (36)
To feel more close to my friends % (n)	19.6 (52)
Place of drug use^a^	
At home % (n)	57.4 (151)
At private parties % (n)	35.4 (93)
Clubbing % (n)	15.8 (42)
During sex % (n)	56.7 (149)
At work % (n)	2.3 (6)
Other % (n)	6.8 (18)
ART non-adherent % (n)	21.2 (56)
Condomless sex % (n)	42.4 (112)
Preforming sexual acts not doing sober % (n)	44.1 (116)
Able to enjoy sex without drugs % (n)	82.9 (218)
Informed physician about drug use % (n)	44.9 (118)
Feel knowledgeable enough about drug use % (n)	73.9 (195)

^a^Multiple-choice question; ART = antiretroviral therapy.

Three percent (9) of participants indicated experiencing side-effects of ART (mostly reported as nausea, vertigo and profuse sweating) consuming ART and drugs and 21.1% (32) said they adjust the dose of ART in preparation for taking drugs, with 49.8% (131) being aware of potential interactions between ART and drugs. Overall 66.7% (176) reported enjoying taking drugs and 73.9% (195) felt their knowledge on drugs was sufficient. Twenty three percent (62) of participants felt that drugs have a negative influence on their life. Not surprising, a significant difference was seen in the univariate analysis whereby polydrug users more frequently reported enjoying taking drugs (77.2% vs 42.5%; p < 0.001) but also more frequently indicated that drugs have a negative influence on their lives (27.7% vs 13.8%; p = 0.024). Polydrug users were also more often non-adherent to their prescribed ART regimens (28.8% vs 3.8%; p < 0.001). In terms of sexual behavior under the influence of drugs, polydrug users reported more condomless sex (51.6% vs 21.3%; p < 0.001) and doing sexual practices they would not do sober (51.4% vs 27.5%; p < 0.001).

### Variables associated with illicit drug and polydrug use

According to the multivariate logistic regression analysis older age, female gender, uncertainty about HIV transmission mode, and residing in a smaller community up to 5000 residents were less associated with drug use (Table 3).

**Table 3:** Characteristics associated with illicit drug use in the study population (N = 438).

Variable^a^	OR	95% CI	p
Age in years	0.96	0.94–0.98	<0.001
Gender (Ref: male)			
Female	0.27	0.14–0.53	<0.001
HIV transmission (Ref: sexual contact)			
IV drug use	1.72	0.65–4.55	0.271
Blood transfusion	0.58	0.09–3.50	0.548
Not clear	0.45	0.24–0.84	0.013
Place of residence (Ref: city with more than a million residents)			
Community up to 5000 residents	0.42	0.20–0.87	0.020
Town up to a 100000 residents	0.48	0.21–1.09	0.081
Large town up to a million residents	0.96	0.41–2.24	0.929

^a^Variables with the cut-off point of p < 0.2 in the univariate analysis were included in the model. During stepwise backwards elimination only those with the significance level of p < 0.05 were retained in the final model. Variables included in the first step were: age, sex, sexual orientation, relationship status, place of residence, mode of HIV transmission, time passed since HIV diagnosis, duration of HAART.

Older age was also significantly negatively associated with polydrug use, while drug use during clubbing, during sex and ART non-adherence were positively associated with polydrug use (Table 4).

**Table 4:** Characteristics associated with polydrug use in study participants who use drugs (N = 265).

Variable^a^	OR	95% CI	p
Age in years	0.97	0.94–0.99	0.030
Highest finished education level	1.36	0.99–1.89	0.062
Country of birth (Ref: Austria)			
EU member state	2.87	0.59–13.78	0.189
Non-Eu member state	0.28	0.06–1.28	0.101
Non European	0.16	0.11–2.37	0.181
Drug use at private parties (Ref: No)			
Yes	1.96	0.89–1.89	0.093
Drug use during clubbing (Ref: No)			
Yes	6.24	1.32–29.52	0.021
Drug use during sex (Ref: No)			
Yes	2.11	1.09–4.05	0.026
ART Adherence (Ref: Adherent)			
Non adherent	8.09	2.19–29.87	0.002

^a^Variables with the cut-off point of p < 0.2 in the univariate analysis were included in the model. During stepwise backwards elimination only those with the significance level of p < 0.05 were retained in the final model. Variables included in the first step were: age, sex, current relationship, time passed since HIV diagnosis, duration of HAART, country of birth, highest finished education level, current CD4+ count known, drug use at home, drug use at private parties, drug use while clubbing, drug use during sex, condomless sex, preforming sexual acts not doing sober, awareness of potential interactions between drugs and HAART, drug non-adherence.

## Discussion

Our study showed the prevalence of illicit drug use among PLWHIV who visit outpatient HIV treatment clinics in Austria to be at 60.5%. This result is threefold higher than the lifetime drug use prevalence in adults in Austria, which is reported at 20.2%, and almost ten times higher than the point prevalence (which was reported at 6.4%) in the general adult population in 2015^9^. Due to differences in sample characteristics as well as investigated periods there is a great variability in reported prevalence between various studies investigating drug use in PLWHIV^26^. For instance Peretti-Watel *et al*. on a large sample of PLWHIV in France report prevalence of 28.6% over the last 12 months, while a recently published study on a Spanish PLWHIV population reports a prevalence level of 44.2% over the last 12 months^27,28^. High prevalence was also reported among AIDS patients^29^.

Illicit drug users in our study population were significantly younger and more often male. Older age and female gender were also found to be associated with less risk of drug use, whereby female participants were four times less likely to report illicit drug use, which was confirmed in similar studies^19,27^ (Table 3). One reason might be the overall lower prevalence of substance use in women, which may be linked to various cultural circumstances^9,30–32^. Furthermore, drug users lived more often in urban areas and in the multivariate model residence in a smaller community was associated with two times less drug use (Table 3). Reasons for this might be easier availability of illicit drugs in larger communities; however, data on this is inconsistent^9,32–34^. Additionally, participants who reported drug use had significantly shorter periods of using ART and living with a seropositive status, which might be explained by their younger age. We also observed an effect of not knowing the exact mode of HIV transmission. Differences in HIV transmission mode concerning illicit drug use have been seen in other studies^27^; however, there is no clear reasoning of this result in literature. One reason might be giving more socially desirable answers or lower educational level found in this group, which was reported to be associated with overall lower drug use^35,36^.

A potential reason for such an overall high prevalence might be due to our study sample, where more than 80% of male participants self-identified as bisexual or homosexual (Table 1). When looking into studies that measured prevalence only in HIV positive MSM, our results are still high but more similar to those findings^11,18,19,35–37^. The ASTRA study, one of the largest studies done on recreational drug use in this population reported a 51% prevalence of recreational drug use in the last 3 months, while Hammoud *et al*. reported illicit drug use in more than half of their participants in the last 6 months^11,19^. Similarly to our result, a US based study reported overall prevalence of 60% in the past 3 months^36^. However, even in MSM focused studies prevalence varies considerably with some studies reporting prevalence at around 20%^35,38^, while a study by Drumright *et al*. reported prevalence at 71.7%^39^.

In terms of most frequently used drugs; cannabis and nitrates (“poppers”) were reported as two most commonly used substances, followed by sildenafil/tadalafil and cocaine (Fig. 1). The most frequently used drug in the general adult population in Austria in 2015 was cannabis, however the reported prevalence is almost 6 times lower in comparison to our study population. Considerable differences were also found in other drugs where 2015 adult prevalence for cocaine, amphetamines and MDMA consumption was 0.4%, which is 29 times lower than in our findings^9^. In a recent Spanish study, Garin *et al*. similarly report cannabis use at 68.5%, sildenafil 28.3%, nitrates 31.5% and cocaine 45.5% among PLWHIV respectively^27^. Additionally, reported use of MDMA in drug users was the same in both our studies. Use of sildenafil and nitrates is contraindicated due to increasing cardiovascular effects^40^, however high prevalence of these substances is reported across studies, with some studies like ours reporting very high prevalence^16,27,39,41^. Drugs associated with “chemsex” and “slamming” (injecting drugs during or before sex); mephedrone, GHB, MDMA and ketamine were also considerably more prevalent in our study in comparison to others^11,27^ (Fig. 1). The prevalence of intravenous use at around 18% is also significantly more than in other studies (Fig. 2)^19,27^. This finding may also be due to a high number of MSM subpopulation in our study sample and as almost 3/4 of our participants indicated using illicit drugs for more sexual stimulation. Both “chemsex” and “slamming” have been identified as practices related to higher risk of condomless sex and HIV transmission in MSM^10,19^. In our study, participants who use illicit drugs noted engaging in condomless sex and in sexual acts they would not do sober in more than 40% (Table 2). Studies indicate that with having good adherence levels almost completely diminishes the possibility of HIV transmission, however there is still considerable risk of HIV transmission among people who are unaware that they are HIV positive or haven’t received therapy long enough to reach HIV suppression. These are the settings where practicing safe sex is still incredibly important^42^.

Using two or more substances during the last 6 months was classified as polydrug use and was found in our study at 42% in the total study population and 69.4% among drug using participants, which is considerably more than other studies^19,27,36,39^. Even though polydrug use is often noted as problematic in PLWHIV, the newest report from the European Monitoring Centre for Drugs and Drug Addiction states polydrug use as problematic in the general young adult population^9^. Differences between mono- and polydrug users were only found in terms of age, where polydrug users were significantly younger than monodrug users and had a shorter time span living with a seropositive status (Table 1). Condomless sex and preforming sexual acts one would not do sober were also significantly associated with polydrug consumption (Table 2). In contrast to other studies that reported an association between increased polydrug use with increased prevalence of condomless sex, or generally showed an association between high risk sexual behavior and illicit drug use^16,19,27,38^, we did not find such an association in our multivariate model. However, polydrug use was significantly negatively associated with older age, and positively associated with drug use during clubbing and during sex (Table 4).

Twenty one percent of our participants reported not being adherent to their prescribed ART regime when using or planning to use drugs (Table 2). Literature shows that adherence is not a stagnant mono-dimensional issue but a dynamic process influenced by many variables. This means that individuals show both times of high and also very low adherence depending on favorability of life circumstances^43^. A meta-analysis of studies on adherence in PLWHIV who use drugs showed an overall optimal adherence in 60% of participants (similar to PLWHIV who do not use drugs) but indicated that studies with a larger investigated time frame showed more variability in adherence than studies with a shorter time frame^44^. Active drug use was found to be associated with adherence problems in other studies as well where non-adherence was also found to be more prevalent in polydrug users, which in our study carried an 8 times higher risk for polydrug use in the multivariant model^19,27,45–47^. Reasons for this may be due to the disruptive influence of illicit drugs on the daily rhythm or due to neural damage that leads into cognitive problems as some illicit drugs cause neural damage and contribute to higher viral replication that causes additional tissue damage^23–25,46^. In terms of ART a particularly high adherence level of over 85% is necessary to achieve viral RNA suppression in patients receiving ART^20^. However, some studies indicated that even though illicit drug use is associated with adherence problems it does not necessarily bring clinical complications^19,27^. In terms of non-adherence and drug use in PLWHIV, most studies focus on unintentional non-adherence because of intoxication^29^. Interestingly, other studies showed purposeful discontinuation with prescribed therapy when planning to use illicit drugs out of fear of potential toxic side effects^48^. However, while potential side effects may possibly frequently occur, more evidence is still needed^21^. In contrast, our participants felt they were knowledgeable on issues concerning drug use and were aware of potential side effects. Nonetheless, one fifth were found to be non-adherent (Table 2), however, this might not lead to clinical manifestations as most of our participants who use drugs reported drug use on a monthly level, which might indicate relatively short bouts of non-adherence (Fig. 2). From a perspective of public health this might indicate that disease based campaigns aimed at increasing individual’s ability to protect themselves may not be effective. Rather campaigns ought to take into consideration the broader social context and help in strengthening and mobilizing community resources. Such approaches have been cited as having a positive role in changing harmful social and cultural norms^49^. This is of particular importance as hard drug users have higher odds of developing AIDS related conditions or dying even after controlling for adherence issues^29,50^.

Finally, study limitation should be addressed. Overrepresentation of male and MSM participants may prevent generalizability of our results, however our study populations is representative of the PLWHIV population in an extramural setting in Austria. Although the response rate was quite good, a selection bias might have occurred, with people taking illicit drugs being more likely to participate in the study compared to non-responders. Using self-reporting questionnaires may lead to reporting bias in terms of wanting to give more socially desirable answers, which even in light of this very high prevalence might still be underreported. Due to logistical issues we did not gather clinical information (CD4 cell counts, viral load etc.) which prohibits their correlation with drug and polydrug use. Additionally, having problems with remembering over the past 6 months may lead to some data distortion. Lastly, cross sectional study design does not allow causal relations.

In conclusion, prevalence of illicit drug use among PLWHIV in outpatient care in Austria is high, with polydrug use being especially commonplace which suggests a large, and until now, an unknown problem in Austria. The results of our study contribute to the growing literature that point to the emerging issues of substance abuse among the PLWHIV. Our results indicate that PLWHIV who use illicit drugs feel knowledgeable about HIV and drug use, which might indicate that public awareness campaigns aimed at increasing knowledge, might not be effective. In light of emerging sexual behaviors such as chemsex and slamming, public health effort should focus on community empowerment and mobilization interventions aimed at younger, urban and male HIV positive people. Further research should focus on identifying fundamental forces that are associated with polydrug use, as well as longitudinal studies that would allow for temporal and causal information.

## Methods

### Participants

Our study participants consisted of patients who visited their chosen HIV outpatient treatment clinic in Vienna, Austria between the 1^st^ of December 2016 and 1^st^ of June 2017. Patient recruitment was done in two centers; one being a hospital-based outpatient clinic and the other an outpatient group practice. In order to minimize potential selection bias, participants were chosen from consecutive patients that came for their regular visits to the outpatient clinic and were asked to participate if they matched the inclusion criteria (over 18 years old, serologically confirmed HIV infection) and were included once informed consent was given.

### Methods

The study was designed as a multicenter cross-sectional study. Due to structural differences between centers, we employed two different ways of gathering data; a standard paper-pencil (in center “A”) and an online based platform (in center “B”). In center “A”, after the consultations with the physician the patients that matched the inclusion criteria were asked to participate in the study. They were explained the study aims and if agreed they were given the study questionnaire to fill out. For the purposes of the study, a room where patients could fill out the survey in privacy was allocated. The patients sealed the questionnaire in an envelope, which was deposited in a locked cabinet by the study team. A member of the study team was present and was able to help with any issues that might have risen during the filling out of the questionnaire. In center “B”, patients, following a consultation, were asked to participate and if agreed they were asked to choose a plain white envelope from a box. Inside the envelope was a letter from the study team with the link to the questionnaire together with a unique code that allowed access. For additional convenience, the questionnaire could have been accessed via smartphone or tablet for which we supplied a QR-code. After the code was used and the participant accessed the questionnaire, the code became invalid, which allowed response rate calculations and prevented multiple entries. The codes were computer generated and there was no way for the study team or the clinic staff to link the individual patients and their code. Also, only the questionnaire answers were saved on a secure server without any personal information. Throughout the online questionnaire, a hyperlink with an email address to the study team as well as a telephone number were available in order to answer any potential questions. After June 1^st^ 2017, the online questionnaire was no longer active and the data containing only answers to the questionnaire were downloaded by the research team. The paper-pencil data were delivered in sealed envelopes and opened by the research team, who had no contact with patients at the study centers and could not link the individual patients to their respective questionnaires.

### Questionnaire

A special questionnaire composed of 31 items divided in 3 parts was created for the purposes of the study. The questionnaire was a combination of single and multiple choice questions as well as open-end questions.

1. HIV-related questions: questions regarding HIV mode of transmission, CD4+ cell count, duration of HIV status as well as type of ART and years spent on therapy used were asked.
2. Questions on drug use: questions concerning types of drugs used, frequency of use, reasons of use, sexual behavior during drug use, personal feelings on drug use, problems when taking ART and drugs, adherence to ART medication, disclosure of drug use to their HIV physician etc. were asked. Participants were asked if they used drugs over the past 6 months and if yes which ones, following by a list of 17 substances that were also written under various slang names in order to improve understanding. Illicit drugs included in the study were: mephedrone, cannabis, methamphetamine, 3,4-Methylenedioxymethamphetamine (MDMA), ephedrine, heroin and other opiates, ketamine, cocaine, lysergic acid diethylamide (LSD), piperazine, amphetamine, benzodiazepines, magic mushrooms, amyl nitrates, tilidine, sildenafil/tadalafil and gamma-hydroxybutyrate (GHB). Self-reported non-adherence to HAART was assessed with the question: “When you use drugs do you skip your HIV medication?”, with participants who answered “yes” and “sometimes” being classified as non-adherent.
3. Socio-demographic questions: questions on socio-demographic characteristics (age, gender, residence, place of birth, level of education, relationship status) of the study populations were asked.

Due to “skip-logic pattern” in both the paper-pencil and online questionnaires the time needed to complete the questionnaire was between 3 and 12 minutes with mean duration being around 7 minutes.

Participants who answered that they used drugs within the last 6 months were classified as drug users; additionally those that indicated using more than one drug within the same period were classified as polydrug users. Furthermore, eleven participants indicated that they used only sildenafil/tadalafil in the past 6 months, which were prescribed by their chosen physician; therefore, these participants were not classified as drug users.

### Statistical analysis

Descriptive statistics were performed for each variable. In case of normal distribution, quantitative variables are shown as mean values and standard deviation and qualitative variables as frequency and percentage. Differences in frequencies of categorical variables were calculated using the Chi-square test and t-test for unpaired samples was used to determine differences between mean values.

In order to determine which variables were associated with drug and polydrug use we performed a multivariate logistic regression model. Based on the results of the Chi-square test and t-test all variables with a cut off value of p < 0.2 were included in the multivariate model. For the model on drug use (Table 3) variables are shown in Table 1, while for the polydrug use model (Table 4) variables are presented in Table 1 and in the text of the results section. We used a stepwise backwards elimination model where the results of the Wald test for individual parameters were examined for each variable. With each regression step, the least significant variable was removed from the model with only those variables associated with drug and polydrug use with a p value under 0.05 being kept in the final model. All p-values below 0.05 were considered statistically significant. The analysis was performed using the SPSS 24.0 statistical software.

### Ethical consideration

The study was approved by the Ethical Committee (EK 16-088-VK) of the City of Vienna on 22^nd^ of June 2016. The study was performed in accordance to the Helsinki Declaration and the principles of Good Clinical Practice. Informed consent was obtained from all participants prior to their inclusion in the study.

### Data availability

The datasets generated during and/or analysed during the current study are available from the corresponding author on reasonable request.
